# Feasibility and Safety of CIOS Spin for One-Stop Pulmonary Nodule Localization and Resection

**DOI:** 10.1093/icvts/ivag069

**Published:** 2026-03-01

**Authors:** Yazhou Liu, Haitao Ma, Xiaojun Yu, Nan Wang

**Affiliations:** The Fourth Affiliated Hospital of Soochow University, Suzhou 215000, China; Department of Thoracic and Cardiovascular Surgery, The Fourth Affiliated Hospital of Soochow University, Suzhou 215000, China; Department of Thoracic and Cardiovascular Surgery, The Fourth Affiliated Hospital of Soochow University, Suzhou 215000, China; Department of Thoracic and Cardiovascular Surgery, The Fourth Affiliated Hospital of Soochow University, Suzhou 215000, China

**Keywords:** pulmonary nodules, preoperative localization, VATS, Cios Spin

## Abstract

**Objectives:**

Ground-glass nodules (GGNs), early signs of lung adenocarcinoma, increasingly require minimally invasive diagnosis due to expanded health screening. This study evaluates the mobile 3-dimensional (3D) Cios Spin system (initially for orthopaedics) in achieving integrated localization and resection of pulmonary nodules.

**Methods:**

This retrospective study analysed 23 consecutive patients who underwent thoracic procedures at the Department of Thoracic and Cardiovascular Surgery, Fourth Affiliated Hospital of Soochow University (Dushu Lake Hospital), from January to December 2023, based on electronic medical records and operative reports. Before surgery, all pulmonary nodules were identified via low-dose or non-contrast chest computed tomography (CT) and confirmed to persist over ≥3 months. The localization needle was precisely placed (needle within ≤5 mm of nodule margin confirmed intraoperatively) with no intraoperative dislodgment. Data collection focused on demographics, procedural details, and perioperative outcomes to evaluate the intervention’s feasibility and safety.

**Results:**

Preoperative localization achieved 100% technical success, defined as accurate placement of the localization needle within ≤5 mm of nodule margin confirmed intraoperatively. Periprocedural complications included one pneumothorax (4.3%) during needle insertion. Positioning exceeded 45 minutes in 2 patients (8.7%) due to anatomical/imaging limitations; 1 (4.3%) required unguided resection without adverse outcomes: Mean durations were 35.3 ± 6.4 minutes for localization and 90.8 ± 20.2 minutes for surgery. All patients recovered uneventfully. No recurrence occurred at 6-month follow-up, but 2-year follow-up is required to assess long-term efficacy (retrospective follow-up ongoing).

**Conclusions:**

This case series supports the feasibility, safety, and clinical efficacy of combining pulmonary nodule localization and resection using the mobile 3D C-arm Cios Spin system. Device/patient factors (eg, system constraints) compromised localization in select cases, necessitating technical refinements and optimized selection criteria.

## Graphical abstract

**Figure:**
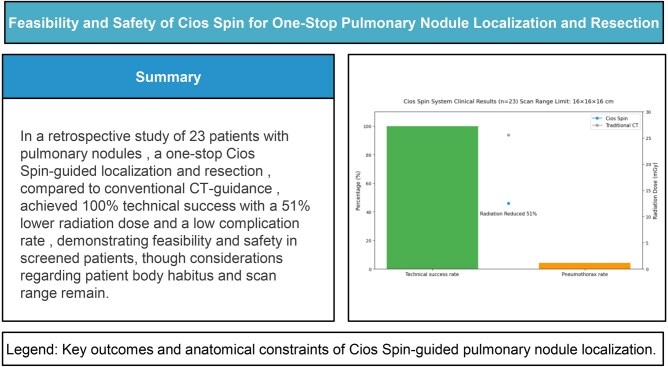

## INTRODUCTION

Emerging localization modalities incorporating image-guided technologies—including flexible trocar systems, microcoil deployment, and indocyanine green fluorescence marking—demonstrate improved safety profiles compared with conventional Hook-wire techniques,^1–4^ particularly enhancing bilateral pulmonary lesion targeting accuracy.^5^ Nevertheless, these advancements fail to alleviate inherent patient anxiety associated with percutaneous interventions under local anaesthesia. Persistent procedural stress underscores the necessity for continued technical refinements in minimally invasive localization workflows, where procedural optimization and psychological support mechanisms represent critical priorities for reducing perioperative morbidity and enhancing treatment adherence.

Previous investigations have established the technical feasibility of intraoperative ultrasound-guided pulmonary nodule identification,^6–8^ although clinical implementation remains constrained by pathoanatomical limitations including severe intrathoracic adhesions and emphysematous lung pathology.^9^^,^^10^ Ultrasonographic resolution proves insufficient for delineating small, low-attenuation ground-glass opacities (GGOs) due to acoustic interference from aerated parenchyma.^9^ The Cios Spin system—a portable cone-beam CT platform—addresses these limitations through high-resolution volumetric imaging (≤0.3 mm isotropic voxels) and intraoperative mobility, enabling real-time 3-dimensional (3D) lesion reconstruction without conventional CT logistical constraints.^11^ This capability proves particularly advantageous for targeting radiographically subtle GGOs inaccessible to ultrasound interrogation. The integrated workflow permits general anaesthesia administration prior to all invasive steps, including CT-guided localization via adjustable needle trajectory optimization under live imaging—enhancing procedural precision while minimizing awake patient discomfort. Despite demonstrated utility in orthopaedic navigation and transthoracic biopsy protocols,^12^ systematic evaluations of its thoracic surgical applications remain sparse, with insufficient reporting on device-specific limitations in pulmonary intervention contexts.

This retrospective investigation employed the Cios Spin mobile cone-beam CT system integrated with percutaneous Hook-wire localization to establish procedural feasibility for preoperative pulmonary nodule targeting. The study protocol was designed to systematically evaluate technical challenges encountered during clinical implementation at our tertiary referral centre, with dual objectives of contributing to standardized application guidelines for this emerging modality and generating evidence-based recommendations for intraoperative workflow optimization.

## MATERIALS AND METHODS

All patients were diagnosed with pulmonary nodules on preoperative chest CT scans, with indications for surgery. This study adhered to the ethical principles outlined in the Helsinki Declaration and obtained approval from the local institutional review board (Ethical approval number: 2024 Ethics Research Approval No. 241043).

### Inclusion of indications

#### Inclusion criteria

1. Radiologically confirmed pulmonary nodules demonstrating radiographic persistence over ≥3 months surveillance, with multidisciplinary consensus (thoracic radiologist and surgeon) indicating resection necessity.
2. Pulmonary nodules located at a depth of >2 cm from the nearest visceral pleural surface.
3. Pulmonary nodules positioned deep within the lung parenchyma, making precise intraoperative localization challenging through naked eye observation, thoracoscopy, and surgeon experience alone.
4. Patients aged 18 years or older who have signed informed consent for surgery.
5. Preoperative assessments ruling out contraindications related to cardiac and pulmonary function and anaesthesia.

According to the Chinese Expert Consensus on the Diagnosis and Management of Pulmonary Nodules (2024 Edition),^13^ the protocol for patients with solitary pulmonary nodules of unknown aetiology and diameter >8 mm is as follows:

Surgical biopsy for diagnosis is recommended (class I recommendation) if any one of the following criteria is met:

1. Positron emission tomography–computed tomography (PET-CT) demonstrates high metabolic activity within the nodule or contrast-enhanced CT shows unequivocal positivity.
2. Non-surgical biopsy results are suspicious for malignancy.
3. The patient, after thorough informed consent, opts for surgical intervention to establish a definitive diagnosis.

#### Exclusion criteria

1. Macronodular (>2 cm) or high-attenuation pulmonary lesions (CT density ≥ −300 HU) amenable to intraoperative visual identification via direct thoracoscopic visualization or manual palpation, precluding the necessity for preoperative image-guided localization during video-assisted thoracic surgical (VATS) procedures.^14^
2. .Nodules situated within anatomical regions demonstrating established segmentectomy or lobectomy indications based on preoperative imaging, precluding the requirement for supplemental localization procedures prior to definitive resection.
3. Nodules occupying juxtadiaphragmatic or subscapular regions, or adjacent anatomical structures where percutaneous localization proves prohibitively challenging due to technical limitations of percutaneous access, elevated procedural risks, or compromised spatial resolution of intraoperative imaging modalities.
4. Individuals aged <18 years.
5. Identification of absolute surgical or anaesthetic contraindications through standardized preoperative evaluation protocols.
6. Suspected severe pleural adhesions, radiologically confirmed calcific deposits, or structural pleural anomalies predicted to compromise localization accuracy under intraoperative imaging guidance.

#### Procedure for localization

Following general anaesthesia induction and double-lumen endotracheal intubation, patient positioning was optimized using preoperative CT-derived anatomical data to align the target nodule with planned percutaneous access trajectories. Standard decubitus positions were selected based on lesion topography. A radiopaque fiducial grid was affixed to the cutaneous surface corresponding to the projected needle trajectory. Ventilation was transiently suspended at end-inspiratory phase under anaesthesiological coordination to minimize respiratory motion artefacts. Initial volumetric acquisition via the Cios Spin system (Siemens Healthineers AG, Forchheim, Germany) confirmed nodule spatial coordinates, enabling triangulation of puncture vectors through the grid’s 3D coordinate system and integrated laser alignment system.

All localization procedures were executed by an attending thoracic interventionist with ≥5 years’ percutaneous pulmonary intervention expertise. Under aseptic protocol, trocar insertion was performed along predetermined trajectories, maintaining a 10 mm parenchymal buffer zone between the localization anchor and nodule periphery to mitigate iatrogenic neoplastic seeding risks.

Following localization, volumetric cone-beam CT acquisition using the Cios Spin system was performed to confirm deployment integrity of the localization anchor, evaluate periprocedural complications, and validate spatial concordance between the marker and target lesion prior to aseptic draping and surgical initiation.

Primary considerations include the following: (1) localization success rate: ensuring the hook-wire released properly and reaches the desired position; the hook-wire should be visible and not detached during the procedure under thoracoscopy; (2) procedure complications: complications related to invasive procedures such as haematoma and pneumothorax during needle localization; and (3) localization procedure duration: Timing commences after induction of general anaesthesia and positioning, continuing until the completion of the entire localization procedure.

### Data measurement

Analyses were executed using SPSS v27.0 (IBM Corp). Categorical variables were expressed as frequencies (%), and continuous variables as mean ± SD with median (interquartile range [IQR]) for non-parametric distributions following normality assessment via Shapiro-Wilk testing.

## RESULTS

**Figure 1.**
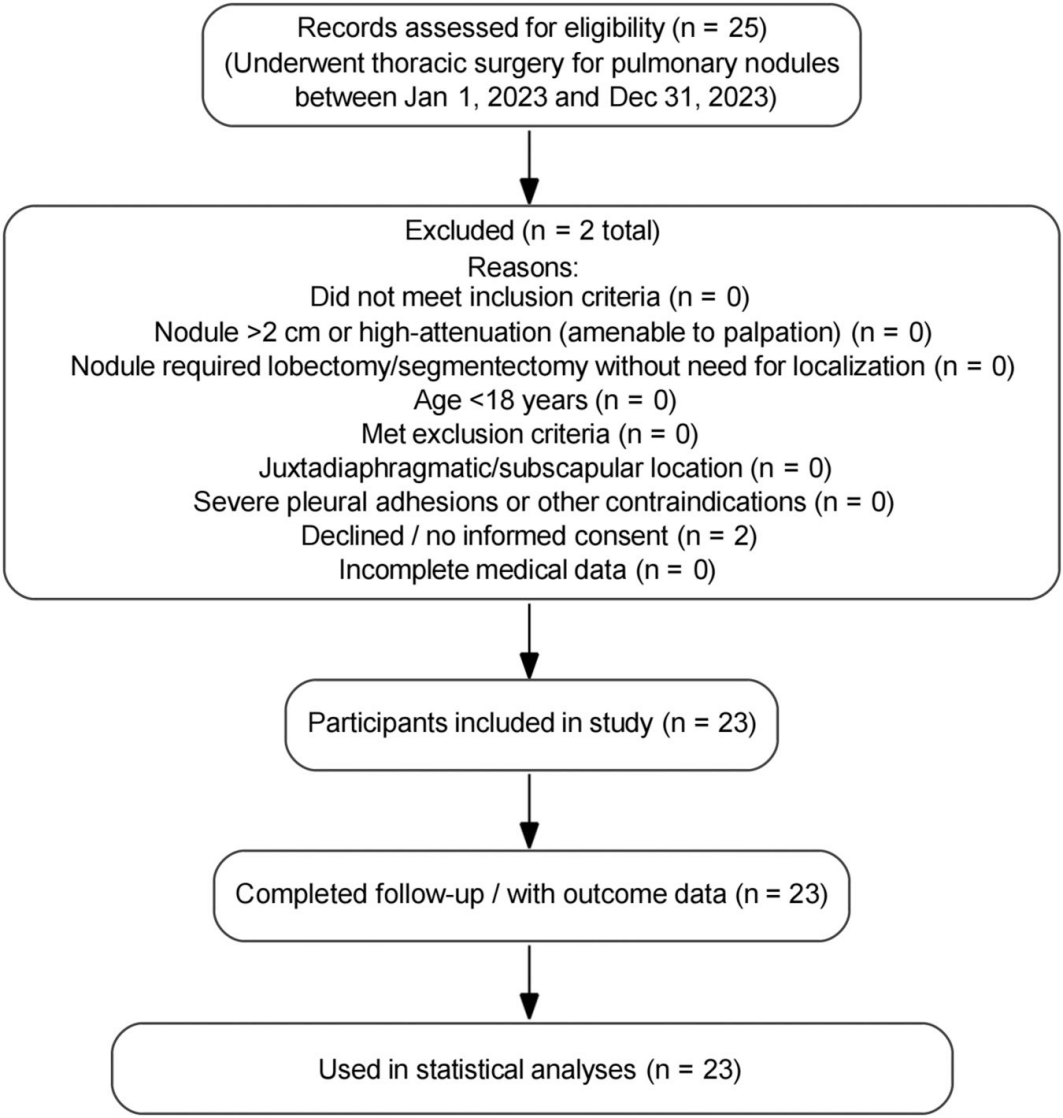
Patient Selection Flowchart

This retrospective study consecutively screened all patients who underwent thoracic surgery for pulmonary nodules at the Fourth Affiliated Hospital of Soochow University (Suzhou Dushu Lake Hospital) between January 1, 2023, and December 31, 2023. A total of 25 patients were identified. After applying the inclusion and exclusion criteria, 23 patients were ultimately enrolled in the final analysis. The detailed process of patient selection is summarized in Study Flowchart (see **Figure 1**).

All subjects achieved technical success in preoperative localization (23/23, 100%). Periprocedural complications included one pneumothorax (4.35%, 1/23), with no additional device-associated morbidity observed. Two cases (8.7%) required extended procedural durations (>45 minutes) due to anthropometric factors and imaging system limitations. One patient (4.35%) exhibited unsuccessful marker deployment under Cios Spin guidance, necessitating intraoperative unguided resection. Thoracoscopic interventions were completed without conversions to open thoracotomy or surgical morbidity in all cases.

Resection modalities comprised wedge resection (12 of 23 cases, 52.17%), segmentectomy (8 of 23, 34.78%), and lobectomy (3 of 23, 13.04%). Histopathological analysis identified chronic inflammation (1 of 23, 4.35%), adenocarcinoma in situ (4 of 23, 17.39), minimally invasive adenocarcinoma (16 of 23, 69.57), and invasive adenocarcinoma (2 of 23, 8.70%).

Localization duration from anaesthesia induction to procedural completion averaged 35.3 ± 6.39 minutes (IQR: 30-39.5 minutes), with total surgical time of 90.83 ± 20.19 minutes (IQR: 75.5-107.25 minutes) (see **Table 1**). All patients achieved uneventful postoperative convalescence and discharge without intervention-related morbidity. Retrospective analysis of 6-month postoperative surveillance data revealed no instances of neoplastic recurrence, metastatic dissemination, or procedure-associated sequelae, including loculated effusions, empyema, or bronchopleural fistulae.

**Table 1.** General Characteristics of the 23 Study Patients

Patient number	Age (years)	Sex	ASA	Tumour size (mm)	Tumour depth	Tumour location	Tumour characteristics	Pathology results	Surgical method	Positioning time (minutes)	Operation time (minutes)
1	36	F	1	6	10	RML	Pure ground-glass	AIS	Wedge resection	35	71
2	45	M	2	8	8	LUL	Mixed ground-glass	MIA	Wedge resection	45	88
3	68	M	2	8	0	RLL	Pure ground-glass	MIA	Wedge resection	23	133
4	56	F	2	10	20	LUL	Mixed ground-glass	MIA	Resection of S4 and S5	25	108
5	47	M	2	6	13	LUL	Pure ground-glass	MIA	Resection of S2	34	89
6	38	F	2	8	16	RUL	Solid	Chronic inflammation	Resection of S1 and S2	45	70
7	57	F	1	9	0	RLL	Pure ground-glass	MIA	Wedge resection	40	107
8	65	F	2	8	0	LLL	Mixed ground-glass	MIA	Wedge resection	39	122
9	64	M	2	6	13	RUL	Pure ground-glass	MIA	Wedge resection	35	119
10	49	F	2	10	25	LLL	Solid	IA	Wedge resection	40	88
11	50	M	2	7	8	RML	Pure ground-glass	MIA	Wedge resection	28	89
12	39	F	1	9	5	RUL	Mixed ground-glass	MIA	Wedge resection	31	66
13	56	F	2	6	4	RLL	Pure ground-glass	AIS	Wedge resection	38	99
14	53	M	2	6	6	LLL	Pure ground-glass	MIA	Resection of S6	27	92
15	47	F	2	5	10	RLL	Pure ground-glass	AIS	Resection of S6	38	79
16	64	M	2	6	28	RUL	Pure ground-glass	MIA	Resection of S1	29	112
17	59	F	2	7	20	RUL	Mixed ground-glass	MIA	Resection of S3	26	101
18	47	M	1	8	6	LUL	Pure ground-glass	MIA	Wedge resection	36	76
19	65	F	2	7	18	RML	Mixed ground-glass	MIA	Right middle lobectomy	39	111
20	48	F	2	6	0	LLL	Pure ground-glass	AIS	Wedge resection	40	76
21	45	M	2	6	10	RUL	Pure ground-glass	MIA	Wedge resection	43	69
22	48	M	1	9	25	RML	Mixed ground-glass	MIA	Right middle lobectomy	39	74
23	38	F	1	10	15	RLL	Mixed ground-glass	MIA	Resection of S6	37	53

Data are expressed as mean ± standard deviation or median (IQR) (eg, age: 52.3 ± 10.1 years; females: 60.9%).

Abbreviation: IQR, interquartile range.

### Limitations

This single-centre retrospective study has a small sample size (*n* = 23), which limits statistical power and precludes subgroup analyses. The lack of a control group and short-term follow-up restrict direct comparison with conventional techniques and assessment of long-term outcomes. Selection bias and the absence of cost-effectiveness analysis should also be considered.

## DISCUSSION

In this study, utilization of Cios Spin for one-stop pulmonary nodule puncture localization indeed offers significant advantages. Using this device, compared with constructing hybrid operating rooms, is cost-effective and facilitates easy equipment mobility.^12^ The Cios Spin workflow resembles conventional CT systems, requiring only specific nodule-targeting spatial/positional adjustments. Its simplicity enhances efficiency and reduces procedural stress, supporting broader clinical adoption.^15^

With standardized training (simulated navigation and real-time guidance), the thoracic team independently operates the system, streamlining workflows and minimizing multidisciplinary dependence.^16^^,^^17^

Compared with the Cios Spin system, VAL-Map achieves multinodule marking via bronchoscopic injection of dye agents, making it suitable for complex anatomical locations,^18^^,^^19^ and new bronchoscopic tools (such as transbronchial pulmonary nodule aspiration, TPNA) may partially alleviate the limitations regarding the diagnostic positive rate. However, this method necessitates additional bronchoscopic procedures,^17^^,^^20^ and dye diffusion may obscure the surgical field.^21^ In contrast, the Cios Spin system offers advantages such as real-time 3D imaging and eliminates the need for patient transfer, although its utility is limited by the scanning range. Future studies could explore integrating VAL-Map with the Cios Spin system to complement their respective technical limitations. The system enables dynamic utilization across available surgical suites, facilitating preoperative nodule localization in diverse operative environments without specialized infrastructure requirements.^16^^,^^22^ This operational flexibility obviates interfacility patient transfers postimaging, streamlining procedural workflows. Integrated general anaesthesia administration prior to invasive interventions minimizes perioperative distress and mitigates marker displacement risks associated with positional transitions.

The Cios Spin system eliminates the need for costly hybrid operating room conversions, as it operates within a standard OR setting with a compatible carbon fibre table. Its acquisition cost is comparable with a conventional CT scanner while saving approximately $500 000 in facility renovation expenses.

Workflow efficiency remains uncompromised compared with conventional localization, yet the system significantly enhances patient experience by avoiding transfers between CT and operating rooms. Localization under general anaesthesia reduces procedural discomfort and anxiety, shortening total perioperative time by at least 30 minutes.

Quantitative assessments revealed marginally prolonged localization durations compared with conventional CT-guided methods, offset by absence of major complications and reduced radiation dose range (6-38 mGy per acquisition;^11^^,^^23^ 50% reduction versus standard CT protocols) (see **Table 2**). Procedural integrity was maintained through intraoperative position retention, achieving 100% needle stability despite extended operational timelines.

**Table 2.** Comparison of Radiation Doses: Cios Spin Versus Conventional CT

Cios Spin (mGy)	a [X]% reduction compared to conventional CT
12.5 ± 3.2^11^	51%

The mean radiation dose for conventional CT was 25.6 ± 5.1 mGy.^11^

Abbreviation: CT, computed tomography.

However, during its application, several limitations of the device have been identified that constrain its clinical utility. Primarily, due to the thickness of the gantry and the need for portability, the working range of the Cios Spin machine is restricted by technical limitations. For successful imaging and localization of pulmonary nodules, certain conditions must be met after appropriately positioning the patient. Specifically, both the target lesion and the skin puncture site must fall within the 16 × 16 × 16 cm isocentre region. If this requirement is not satisfied, it becomes impossible to simultaneously visualize the puncture site and the target lesion, thereby hindering the planning of an accurate puncture path.

Thus, this technique is suitable for solitary nodules located outside the subdiaphragmatic/subscapular regions and with a body thickness of ≤38.9 cm. For obese patients or nodules in complex locations,^24^ other techniques (such as intraoperative ultrasound) may need to be combined.

The skin puncture site (point A) meets the upper and lower surfaces of the carbon bed at points B and C, respectively. Based on the Pythagorean theorem, the square of length AB must not exceed the square of the Cios Spin arm’s maximum diameter minus half the square of the bed width. For example, with a bed width of 52 cm and a machine arm diameter of 93.6 cm, AB must be ≤38.9 cm and AC ≤46.8 cm. Failure to meet these criteria may cause gantry-bed collision during rotation, resulting in imaging failure.

Some patients with larger body sizes or obesity may attempt to adopt a lateral 45° supine or prone position to meet procedural requirements; however, certain lesions may remain inaccessible due to limitations in positional adjustments or body size, rendering localization unfeasible. Furthermore, the machine’s angular and imaging constraints create blind spots between the equipment and the operating table, making it particularly challenging to scan specific areas such as the lung apices. In cases where localization proves impossible, it may become necessary to abandon the procedure and proceed directly to surgery or transfer the patient to a hybrid operating room for remedial measures. Such adjustments not only prolong preoperative preparation time but also increase surgical complexity and associated risks. Additionally, the scanning coverage of the system is significantly smaller than that of traditional CT scans, with each scan encompassing an area of only approximately 16 × 16 × 16 cm. Misalignment of the device during the initial scan may result in the lung nodules being positioned outside the scanning range, requiring repositioning of the machine. This increases the time required for localization and reduces overall surgical efficiency. Finally, to prevent excessive metal artefacts caused by conventional metal operating tables, which can severely compromise imaging quality, the use of Cios Spin for lung nodule localization necessitates a compatible carbon bed. While carbon beds can be quickly repositioned using specialized transfer equipment within the surgical area, the limited availability of such beds may hinder the localization process unless multiple fixed or movable carbon beds are readily accessible.

Moreover, the scanning speed of the Cios Spin is relatively slow, with each scan requiring approximately 30 to 60 seconds depending on the initial parameter settings. During the scanning process, it is recommended that the anaesthesiologist temporarily pause ventilation or reduce the ventilation rate, while continuously monitoring the patient’s oxygen saturation to ensure safety during the respiratory pause. In cases where the patient maintains spontaneous breathing or exhibits a high ventilation rate, the slow scanning speed may introduce additional artefacts, thereby compromising imaging quality. Even when mechanical ventilation is fully suspended, the imaging quality remains inferior to that of traditional CT scans, making it challenging to accurately identify low-density pure ground-glass nodules.^25^

To address these challenges, the following improvement strategies are proposed: First, it is critical to conduct preoperative screening for patients scheduled for localization. Moreover, future initiatives will focus on collaborating with manufacturers to develop an upgraded model with an expanded scanning range (eg, 20 × 20 × 20 cm),^11^ specifically tailored to accommodate obese patients. Patients with excessive obesity or large anterior-posterior or transverse thoracic diameters may have target nodules located beyond the scanning range, rendering the localization procedure unsuitable for the equipment. Preoperative CT-based planning (trajectory measurements and feasibility assessment) is mandatory for all patients. If conventional positioning fails to align the nodule within the imaging range (16 × 16 × 16 cm), a 45° lateral tilt (supine/prone) may be required. This approach minimizes the likelihood of altering the localization strategy during the procedure. Additionally, preoperative 3D reconstructions should be utilized to measure the linear distance from the nodules to the lung apices, and the approximate location of the nodules on the body surface should be determined beforehand. This enables early planning for optimal equipment placement, thereby improving procedural efficiency. Finally, the Cios Spin system for preoperative nodule localization is a novel technique with limited global studies. Based on a systematic literature review, existing research (e.g., Hsieh et al^12^) reports sample sizes of 20 to 30 cases. To align with these precedents and contribute timely insights, publication of findings after initial case accrual was prioritized.^12^ As more procedures are performed, the cohort will be expanded and additional cases supplemented.^26^

## CONCLUSION

**Figure 2.**
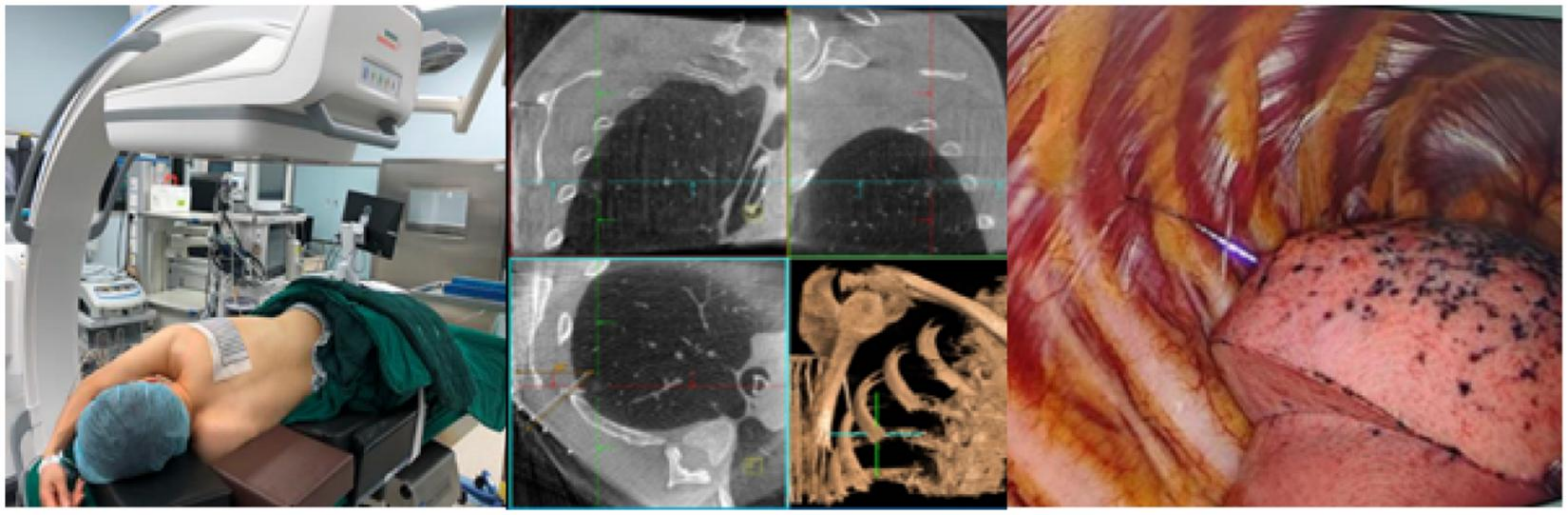
Workflow of One-Stop Nodule Localization and Resection Using the Cios Spin System. Left panel: Preoperative patient positioning and planning of the scanning region. Middle panel: Localization of the pulmonary nodule and determination of the puncture depth and angle based on CT imaging (see **Figure 2**). Right panel: Intraoperative visualization during VATS surgery, demonstrating precise localization with the needle accurately positioned at the target site (see **Figure 2**). Abbreviation: VATS, video-assisted thoracic surgical.

**Figure 3.**
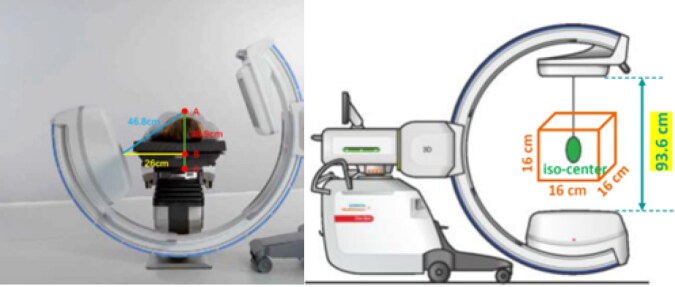
System Limitations and Patient Adaptations. Left panel: The square of the length of line segment AB must not exceed the difference between the square of the maximum diameter of the Cios Spin CT arm entrance and half the width of the carbon bed squared (see **Figure 3**). Right panel: Both the target lesion and the skin puncture site must be located within the 16 × 16 × 16 cm region centred at the iso-centre (see **Figure 3**). Abbreviation: CT, computed tomography.

The Cios Spin mobile cone-beam CT system supports clinical viability for preoperative pulmonary nodule localization by mitigating procedural distress and anxiety associated with percutaneous interventions under local anaesthesia while maintaining targeting accuracy. This technology introduces a novel image-guided thoracic workflow, streamlining traditional CT-based methods. However, its clinical adoption requires comprehensive preoperative planning to overcome system limitations in imaging range/resolution. Refinements in patient selection and procedural algorithms are key for wider use (**Figures 2 and 3**).

## Data Availability

All data are incorporated into the article.
